# Employees’ Green Enterprise Motivation and Green Creative Process Engagement and Their Impact on Green Creative Performance

**DOI:** 10.3390/ijerph19105983

**Published:** 2022-05-14

**Authors:** Xiao Hu, Shumaila Mazhar Khan, Shijiao Huang, Jawad Abbas, Mirabela Constanta Matei, Daniel Badulescu

**Affiliations:** 1 School of Economics and Management, Wuhan University, Wuhan 430072, China; 2 Faculty of Management Sciences, National University of Modern Languages, Islamabad 44000, Pakistan; shumaila.awan@numl.edu.pk; 3 School of Intercultural Studies, Jiangxi Normal University, Nanchang 330022, China; huangshijiao@jxnu.edu.cn; 4 Faculty of Management Sciences, University of Central Punjab, Lahore 54000, Pakistan; jawad.abbas@ymail.com; 5 Department of Management-Marketing, Faculty of Economic Sciences, University of Oradea, 410087 Oradea, Romania; mmatei@uoradea.ro; 6 Department of Economics and Business, Faculty of Economic Sciences, University of Oradea, 410087 Oradea, Romania; dbadulescu@uoradea.ro

**Keywords:** green motivation, green creative processes engagement, green creative performance, sustainability

## Abstract

Based on the recent surge in environmental degradation issues mainly caused by the manufacturing industry and the inadequacy of the measures taken to respond to them, this research focuses on investigating whether employees’ motivation to protect the natural environment leads to their green creative performance (GCP) at work. It also examines the role of green creative process engagement (GCPE) as a mediator between green motivation (GM) and GCP. Structural equation modeling (SEM) is used to examine the hypotheses which indicated that employees’ GCP appears to be significantly influenced by their GM. Moreover, GCPE is also found to act as a mediating factor between the two. Moreover, industry type and gender are found to play significant roles in the studied variables. The current research is among the pioneer studies that focus on involving employees in the pro-environmental creative process through green motivation, leading towards GCP, an essential element for achieving the United Nations Sustainable Development Goals. The study’s findings can help companies promote GCP to solve today’s most pressing environmental issues and achieve sustainable development goals.

## 1. Introduction

With each passing day, the world becomes more aware of environmental concerns, most of which are caused by manufacturing industries, which are becoming more prevalent [1]. Environmentalists are constantly raising awareness about the depletion of natural resources and forcing businesses to look for alternative renewable and sustainable energy resources [2]. As the world’s largest green environment initiator, the United Nations Global Compact (UNGC) enforces environmental sustainability principles and helps achieve sustainable development goals (SDGs) [3]. Sustainable development (SD) has become a significant concern for academicians, industrialists, and governments worldwide [4]. Businesses must ensure sustainability at all levels of their operations, especially regarding operations, resource usage, waste disposal, and environmentally friendly processes like recycling [5]. Businesses worldwide have begun to prioritize SD practices, reducing their reliance on nonrenewable energy sources and implementing eco-friendly policies to meet their sustainability objectives [6].

Individual employees have been motivated to shift their mental schemata of affective (motivation) and cognitive (knowledge of the environment) resources from a traditional inward perspective toward a more contemporary outward-oriented outlook while causing minimal harm to the natural environment [7]. This shift is caused by environmental degradation awareness [8]. Employees play a vital role in the success of an organization because they are the driving force behind the entire value chain [9,10]. The creative performance of employees is studied to be linked with two important variables, namely individual creativity (IC) and innovative work behaviors (IWB) [11]. IC is the generation of a beneficial idea whereas IWB are the behaviors towards the implementation of such ideas [12]. When the creative performance is ensured, keeping in view the sustainable perspective, green creative performance (GCP) results [8]. In other words, to achieve the GCP target, the firm must realize the need to incorporate green creativity (GC) and green innovative behavior (GIB) as its foundations. GCP can help an organization gain and maintain a competitive advantage by enticing employees to adopt green practices in the workplace [13].

Green creative employees develop innovative solutions to environmental problems, help spread those solutions to others, and devise strategies for putting them into action [8]. Different terms for environmental performance have been used to describe the subject, including corporate sustainability [14], environmental performance [1], and green creative performance [15]. To reap the benefits of green initiatives and generate GC, organizations combine sustainability concerns with a creative mindset [16]. There is a solid need to enrich the literature on GCP and its drivers since this phenomenon is introductory [17].

The knowledge about green performance and pro environmental behaviors leads to an enhanced level of green motivation (GM) to materialize GCP [18]. The GM of employees is indicative of their support of and concern for the environment; it is a vital force ensuring green environmental performance [1]. Much of the existing work reports the motivational drivers of an individual to engage in pro-environmental behavior [19], and current research is focused upon studying the outcome of GM in realizing GCP. Based on what influences individuals for green creativity, Li et al. [20] divided green motivation (GM) into two groups, namely green intrinsic motivation (GIM) and green extrinsic motivation (GEM). GIM is a person’s ability and willingness to engage in green behaviors based on an internal locus of causality, such as doing things that conserve the environment out of love for the natural world [21]. Organizational green goals are more likely to be met if employees’ green values are aligned with those of the organization [22]. On the other hand, GEM is governed by external stimuli, such as depicting green behaviors and actions to be rewarded or receive approval from others [20]. Employees are more likely to be motivated by a company’s green HR practices if they are paid in accordance with a green compensation plan or a green performance appraisal plan [23,24]. GIM includes behaviors such as preferring green products over non-green ones, minimizing environmental damage by using recyclable products, conserving water and electricity, and so forth [25]. On the other hand, GEM refers to environmentally friendly behaviors like waste reduction, efficiency improvement, and environmental conservation due to some external monetary or non-monitory benefit. However, research has shown that the effect of GEM is shorter than the inherent motivation [26]. Extrinsic motivation is viewed as reducing one’s ability to be creative and innovative [27].

An organization’s employees are more likely to participate in green creative processes and activities when green management and approaches are given priority [18]. Kalyar et al. [15] first proposed the term “green creative processes engagement,” or GCPE, in 2021. It is an employee’s involvement in the methods or processes involving identifying the problem, searching for information, and generating ideas for the GCP of an organization.

A few researchers have studied employees’ pro-environmental behavior and creative performance from a different perspective. For instance, Li et al. [20] stated that green transformational leadership significantly predicts green motivation among employees. Mahmood et al. [28] highlighted the role of transformational leadership in employee creative process engagement. They said that top management significantly reshapes employee attitudes and gets them involved in the creative process. However, the authors could not find any study that highlights employees’ GM role in engaging them in pro-environmental creative processes, a precondition for their GCP. Based on the green theory and the conservation of resources theory, this is among the pioneer studies that link employee green motivation with their engagement in pro-environmental creative processes, which leads to GCP. Thus, the purpose of this study is to find out whether: (1) green motivation improves the green creative performance of employees; (2) green motivation affects employees’ green creative process engagement; and (3) green creative process engagement mediates the relationship between green motivation and green creative performance.

Current research adds to the body of knowledge about the links between GM-GCP and GM-GCPE by explaining the extent of GM of employees leading to GCPE and the GCP, which is scarce in the literature. The second point is that the inclusion of GCPE as a potential mediator between GM and GCP explains the fundamental phenomenon of whether GM improves GCP through the mediating role of GCPE among employees in manufacturing and service firms. Thirdly, it takes contextual factors, such as gender and the nature of the industry, as control variables to understand whether these factors significantly affect the principal findings or not. This research follows a multivariate statistical technique and structural equation modelling (SEM) approach to achieve its objectives, strengthening its contribution to the literature. Overall, this study contributes empirically and conceptually to the existing body of knowledge by developing and validating a model of the antecedents of GCP in the manufacturing and services industry.

## 2. Theoretical Foundation

### 2.1. Green Theory and Conservation of Resources Theory

Since the advent of the industrial revolution, industries’ consumption of natural resources, especially the manufacturing ones, has risen significantly [29]. These businesses rely heavily on nonrenewable energy sources and pollute the various aspects of the environment in various ways, including water, air, and soil [30]. It has posed a serious question on the adequate availability of natural resources for future generations. Different stakeholders have raised their voices to raise awareness about diminishing natural resources and promote environmentally friendly activities in response to this situation. Creative performance of the organizations is increasingly driven by environmental considerations [7]. Green theory, a multidisciplinary approach proposed by Eckersley [31], explained the rise of green theorizing in social sciences and its prevalence in local, national, and international contexts with global outlooks. As per this theory, environmental sustainability must be ensured at all levels. Moreover, a company’s competitive position can only be maintained by balancing the interests of all the stakeholders involved, including society and nature [32].

**Figure 1:**
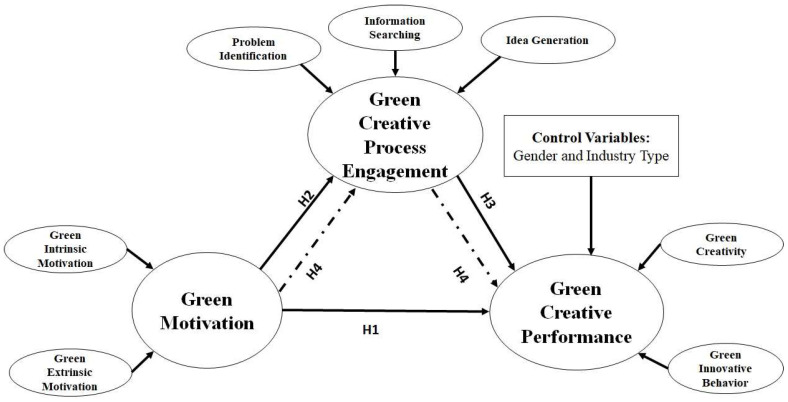
Conceptual Framework.

GCP’s roots can be traced back to green theory. Individual creativity (IC) and innovative behaviors (IB) were studied by Mutonyi et al. [12] as a two-dimensional concept of GCP. Based on the conservation of resources (CoR) theory by Hobfoll [33], employees’ resources, like GM, help them engage in GCP and deal with challenging situations like the achievement of GCP, leading to the accumulation of additional resources. Companies can gain a long-term competitive advantage by utilizing scarce, valuable, and non-replaceable resources. The study’s conceptual model can be seen in Figure 1.

### 2.2. Green Creative Performance

A growing number of people worldwide are responding creatively to SD initiatives [8]. Despite its difficulties, its significance as a catalyst for long-term change cannot be understated. The 2030 Agenda for Sustainable Development of the United Nations (UN) and UNESCO has emphasized that creative solutions to sustainability issues must be applied globally by leading organizations [34]. According to the principles for responsible management education (PRME), the UN has made an effort to include creativity in business schools to generate sustainable value for the future [35]. Due to different stakeholder pressures, organizations have started allocating proportionate resources to environmental development initiatives. Firms strive to conserve their employees’ precious and rare resources to achieve GCP and give their company a long-term competitive advantage [36].

The rise in a person’s GC and GIB can be explained by GCP [12]. Ideas for new and valuable green products, services, or processes are the primary focus of GC [2]. Environmental innovation indicates how organizations are progressing toward greener practices [37]. Environmental problems and degradation necessitate finding new solutions, so GIB searches for novel methods, technologies, and plans to implement new ideas [38]. Adopting, implementing, or using the creative idea shows new ways of achieving SD goals.

Employees at GM are more likely to come up with innovative solutions to reduce the amount of paper, water, electricity, and other resources used at work [1]. They are driven to develop products and services that reduce environmental damage due to business operations by their innate concern for the environment [26]. Green production and GCP cannot be ensured if companies do not have the knowledge and commitment of their employees to go green. As a result of GIM, employees are proud to work for a company committed to environmental responsibility [20]. GCP will improve with the increase in GIM-trained employees. Individual, social, and organizational factors on green information technology (GIT) were examined by Ojo et al. [25], and a positive relationship was found between green beliefs, attitudes, and practices. Gilal et al. [21] also concluded that the green values of employees strongly influence environmental performance.

Similar to GIM, GEM exercises the motivation to be involved in suggesting or implementing a green idea for any monitory or non-monitory gain [1]. The GEM of employees results from a company’s adoption of green human resource practices, including green compensation and green performance management. The authors claim that employees motivated to protect the natural environment tend to look for beneficial and environmentally friendly ways in the current research. Therefore, it is recommended that:

**Hypothesis** **1** **(H1).** *Green motivation has a significant positive impact on employee green creative performance*.

### 2.3. Green Creative Processes Engagement 

GCPE involves involving employees in problem-solving activities such as identifying environmental issues, gathering the relevant data, and developing creative solutions to address environmental issues [15]. Participation in eco-friendly creative processes is necessary for protecting the natural environment and its resources. It ensures that GC’s iterative processes are understood. Employees with higher GM, particularly GIM, have an inner love for the environment that forces them to engage in processes to identify alternative solutions to environmental issues or generate creative ideas to solve them [26]. In addition to those with GEM, those with GCPE are also interested because their company offers incentives for green performance [39]. As a result, they are more motivated to solve their problems [40].

GM enables people to identify information about the environmental problem, enhancing their ability to correctly understand the problem’s nature and causes [8]. They can generate a wide range of environmentally-friendly ideas because of their comprehensive knowledge [1]. The CoR theory states that when employees with GM are confronted with an environmental issue, they are more likely to develop an innovative solution that uses the least available resources possible [33]. To do so, they must engage in a rigorous creative process. Based on green theory and CoR theory arguments, this study claims that GM encourages people to engage in environmentally-friendly creative processes. Therefore, it is proposed that:

**Hypothesis** **2** **(H2).** *Green motivation is a significant predictor of employees’ green creative process engagement*.

### 2.4. Green Motivation, Green Creative Processes Engagement, Green Creative Performance

CPE is regarded as an essential step in the creative process. It consists of various components, each contributing to an increased IC level [41]. From an environmental perspective, GC’s antecedents are distinct from general creativity, and the mechanism of GCPE and its impact on GC, GIB, and GCP requires further study. As part of its mission, the GCPE works to identify environmental issues, find information to help preserve the planet’s natural resources, and develop solutions that minimize waste and promote recycling [42]. This leads to a person proposing novel and valuable solutions to sustainability issues due to GC involvement [15].

The GIB of an employee denotes suggestion and initiation, application, and commercialization of the novel and workable idea for the environmental problem [43]. It requires going beyond the basic job requirements by searching for new techniques or product ideas to challenge the established routines, championing the idea to others, developing plans, and securing funds to implement the idea. Therefore, GCPE, at the first stage, ensures that the employee has structured the problem well; secondly, it leads a person to search and encode the relevant information. Finally, it fosters a new understanding of the problem. Time spent on each subsequent stage of GCPE is thought to impact the quality and originality of the solution [44]. Thus, it is stated that GCP is positively influenced by the GCPE, including GC and GIB. Subsequently, the following hypothesis is proposed:

**Hypothesis** **3** **(H3).** *Green creative process engagement has a significant positive impact on green creative performance*.

A higher level of concern for environmental protection, i.e., improved GM, encourages the generation of high-quality information for regulating an employee’s behavioral function in relation to environmental problems and solutions ([45]). Because of this, employees are engaged in creative processes that include environmental problem identification, information search, and pro-environmental creativity, all of which contribute to an improved GCP in accordance with the perspective of CoR theory. As a result, it is proposed that:

**Hypothesis** **4** **(H4).** *Green creative processes engagement mediates the relationship between green motivation and green creative performance*.

## 3. Research Methodology

### 3.1. Methods 

Pakistan’s Securities and Exchange Commission (SECP) is considered the country’s most reliable business directory, and this study is focused on companies listed on the SECP. Service firms, including business consultations and advertising agencies and manufacturing firms including medicine companies and fast-moving consumer goods (FMCGs) were targeted. Moreover, only firms with the certification or having the intention to apply for environmental certification (ISO 14000) were targeted to collect data. Information was gathered from employees at all levels, from entry-level workers (non-managerial) to those at the top of the corporate chain (top management). Likert scales of one to five were used to rate the participants’ GM, GCP, and GCPE.

To approach firms, non-probability purposive sampling and non-probability convenience sampling techniques were used for employees. Google Forms, email, and personal visits were used to distribute questionnaires. A total of 501 questionnaires were distributed among firms from June 2021 to October 2021, out of which only 257 were returned, resulting in a response rate of 51.3%. Of the 257 responses received, 43 were discarded because they were incomplete or incorrectly answered. Moreover, 97 responses were also collected via Google forms, making 311 usable responses for the final analysis. Out of the total useable response, 188 responses were received from managerial position holders, such as middle, junior, and top-level managers and 123 responses were extracted from non-managerial staff. Similarly, 138 responses were received from service firms and 173 from manufacturing ones. From a gender perspective, 65.27% of responses were received from male workers and 34.7% from female staff members. Table 1 explains the detailed demographic information of the respondents.

Participants in this study were asked to complete two sections of the questionnaire, the first of which contained questions about their demographics and the second focused on the study’s variables. The items for GM containing GIM and GEM were taken from Guay et al. [46] and Junsheng et al. [18], respectively. GCPE is measured using the Kalyar et al. [15] scale. The items for CP constituting GC and GIB were adapted from Soda et al. [47], and Luu and Scot [8,48]. The first 50 responses from Islamabad-based companies were subjected to a pilot study to ensure the adapted items’ reliability and validity. The initial results showed an internal consistency range of 0.86 to 0.93. Hair et al. [49] proposed a minimum of 0.7 for this range. A comprehensive survey was launched in light of the pilot study’s findings.

Structural Equation Modelling (SEM) is used to examine the relationship between GM, GCPE, and GCP. This technique effectively examines the relationship between latent and observed constructs, and the collected data were analyzed using AMOS v.25 and SPSS v.25. Sample size, common method bias (CMB), and multicollinearity aspects were examined to ensure that the data were robust enough for factor analysis. According to the Kaiser-Meyer-Olkin (KMO) test, the sample size was adequate, with a value of 0.907, which is in line with the minimum requirement of 0.6 proposed by Kaiser and Rice [50]. The variance inflation factor (VIF) was used to test for multicollinearity. The resultant value of 1.163 is well within the upper limit of 4, indicating that multicollinearity is absent [49,51]. CMB is the variance when independent and dependent variables are analyzed using the same research field. For this concern, the researcher used Harman’s single factor test, and the result was 40.01 percent, which is less than the maximum limit of 50 percent.

### 3.2. Results

Hinkin [52] recommended that confirmatory factor analysis (CFA) be performed to ensure that the study’s measurement model was valid and unidimensional. An observational dataset was subjected to CFA to determine the relationship between latent and visible variables. Composite reliabilities of the measurement model and Cronbach’s alpha values of the constructs were checked (refer to Table 2), which fulfilled the lowest requirement of 0.7 by Molina et al. [53]. This means that the items are internally consistent and are constantly measuring the same construct. Convergent and discriminant validity was measured to ensure the validity of the model. Convergent validity is when the measures that should be related are related in reality and can be judged by the factor loadings of the measure. All of the factors were loaded at more than 0.7, indicating the convergent validity of the indicators [54], explaining that the items measuring a construct are similar to each other. An AVE value greater than 0.5 also confirms the convergent validity recommended by Molina et al. [53]; thus, the study constructs fulfill this condition.

Discriminant validity means that the measures that should not be related are discriminant in reality, i.e., each measurable construct is conceptually or empirically discriminant from other constructs. For this, Hair et al. [49] stated that each pair of predictor variables must correlate to less than 0.9. As mentioned in Table 3, all correlation values meet this requirement. According to Fornell and Larcker [55], the variance in a construct is caused by its indicators, or similar indicators are converging on the same construct to authenticate discriminant validity. Table 3 indicates that all instruments meet the reliability and validity requirements.

A model’s fit can be judged using seven commonly used indicators, including chi-square to the degree of freedom (x^2^/df), the goodness of fit index (GFI), the adjusted goodness of fit index (AGFI), comparative fit index (CFI), normative fit index (NFI), root mean square error of approximation (RMSEA), and standardized root mean square residual (SRMR) [56]. The Tucker-Lewis index (TLI) measurement and the structural model fit are further ensured in this study. The measurement model’s x^2^/df is 1.164, and for the structural model, its value is 1.191. Both values lie within the maximum limit of 3.0 proposed by Byrne [57] and the maximum limit of 2.0 recommended by Bagozzi and Yi [58]. Likewise, the value of NFI, GFI, AGFI, CFI, and TLI are 0.926, 0.911, 0.927, 0.942, and 0.954 for the measurement model and 0.931, 0.922, 0.951, 0.956, and 0.951, respectively, for the structural model, which are all well above the minimum limit of 0.9 as suggested by Bollen [59]. Furthermore, values of RMSEA and SRMR are 0.029 and 0.0352 for the measurement model and 0.034 and 0.0331, respectively, for the structural model, which is much lower than the maximum ceiling of 0.08 suggested by Browne and Cudeck [60] for RMSEA and Hu and Bentler [61] for SRMR. Therefore, both measurement and structural models (refer to Table 4) greatly fit the collected data.

The proposed hypotheses were examined using SEM. For H1 a positive and significant impact was found by GM on the GCP of employees, with β and *p*-values of 0.294 and 0.003, respectively, i.e., employee green motivation has a significant positive impact on their green creative performance to be accepted. For H2 the direct effect of GM on GCPE was checked, and the results showed that GM is a significant positive predictor of GCPE with a *p*-value of 0.003 and a β value of 0.301, which led to the acceptance of H2. Further, to check H3, the link between GCPE and GCP was examined, which presented a β value of 0.3487 and *p*-value of 0.01, pointing towards the acceptance of the hypothesis, ensuring a significant positive impact of GCPE on employee GCP (refer to Table 5). For H4, the impact of GM on GCP was reduced from 0.294 to 0.226, with a significant *p*-value of 0.034 and composite reliability of 2.198. The impact of GM is reduced because some of the effects of GM are transferred to GCPE. Based on these results, it is inferred that GCPE partially mediates the link between GM and GCP, as the results are still significant [54]. Bootstrapping was also performed to reconfirm the mediation effect. With 1000 bootstrap samples and 95% bias correction, the direct effect of bootstrapping exhibited a value of 0.352 with a *p*-value of 0.021, whereas the indirect effect of GM on GCP through GCPE exhibited a bootstrapping result of 0.264 and a *p*-value of 0.029. As the value of indirect and direct effects are significant, it substantiates the partial mediation of GCPE within the relationship of GM and GCP (Table 5). Hence, H4 is also accepted.

This study contains two control variables, i.e., industry type and gender. The inclusion of industry as the control variable indicated a significant result. This means that employees’ motivation to protect the natural environment varies from industry to industry. Employees from the manufacturing sector including medicine companies and fast-moving consumer goods (FMCGs) were found to be more motivated for eco-friendly innovative ideas and solutions. Moreover, considering gender as a control variable, female workers depicted more involvement in environment-friendly activities and ideas than male workers. This means that female workers are more motivated to protect the natural environment and more willing to follow eco-friendly practices as compared to their male counterparts.

## 4. Discussion, Research Implications, and Limitations

### 4.1. Discussion

This research examines the relationship between employees’ GM and its impact on their GCP. Considering the significance of the creative process, GCPE is taken as a mediating variable. The authors focused on the manufacturing and services firms in emerging economies in Asia, specifically Pakistan. As per the findings, employees’ GM is a significant predictor of their GCP. GCP can be improved if employees are motivated to protect the environment and resources internally and externally. This result matches Li et al.’s [20] and Abbas and Dogan’s [62] study that employees with GIM are more likely to engage in environmentally friendly behaviors than others. According to Ahmed et al. [1], employees with GM try to find a creative solution to environmental problems, which is in line with the empirical findings of our study. GM of employees to protect natural resources from their activities and those of the organization’s operations by reducing resource waste can lead to GCP to enhance organizational competitive advantage.

A company’s image as a responsible corporate citizen can be enhanced by GCP-educated employees, making it a more attractive option for customers. There are many similarities between GM and GCP, which focus on improving environmental performance and reducing waste. GM can increase employee involvement in environmentally friendly initiatives through more informed participation. Thus, organizations must strive for long-term success by enhancing the GM of their workforce through green human resources and related strategies. By doing this, companies can get their employees involved in conserving natural resources, reducing hazardous waste emissions, and improving the organization’s commitment to environmental stewardship.

The analysis of the relationship between GM and employee GCPE also presented a significant positive relationship. This means that employee GM is a significant predictor of their GCPE and matches Li et al.’s [20] findings that employees with GM tend to get more involved in green creative processes than others. Ali et al. [26] also suggested that employees with GIM tend to participate in innovative activities at a higher rate than others. GM can increase employee involvement in environmentally friendly initiatives by empowering them with more information and training. As a result, businesses should make an effort to increase employee involvement by providing green compensation and setting up green training programs. The organization’s support and encouragement will help employees understand and comprehend environmental issues, improve their search for information, and help them select the most appropriate solution.

Furthermore, it was found that GCP is significantly influenced by GCPE. Researchers such as Cheung et al. [41] and Li [63] have shown that creative process engagement is linked to creativity, and this finding is consistent with their studies. As soon as a worker discovers an issue that is harming the environment and seeks information that can reduce waste, it is more likely that they will make an effort to develop an innovative and valuable solution. As a result, they propose a concept. Still, they also attempt to commercialize that concept into a workable plan by raising the necessary funds, investigating new techniques, and promoting the concept to others. The knowledge base is scarce on the role of GCPE as a primary intervening mechanism between GM and GCP. The results showed positive and significant results of the mediating role of GCPE within this relationship, which means that GM can directly and indirectly (through GCPE) affect GCP or GCPE functions as a mediating factor for the nexus between GM and GCP.

### 4.2. Study Implications

#### 4.2.1. Practical Implications

Managers can use the findings of this study to help foster their employees’ GCP. To stay ahead of the competition and enhance their company’s reputation, organizations should implement GCP, according to the green outlook. In addition, the United Nation’s focus on sustainability has made environmental standards compliance imperative for organizations. Accordingly, this study recommends that the management of an organization trying to improve the GM of its employees fosters not only the GC but also the GIB, which ultimately enhances GCP. Green human resource practices, such as green hiring, green training and development, and green compensation can help foster GEM and build resources for GIM to participate in green initiatives and engagements. This commitment should come from management and leadership. GM encourages employees to participate in green activities, which improves GCP. The program serves employees’ desire to protect the environment and the company’s image. A company’s intellectual capital can be boosted by employing environmentally conscious people who perform well in an eco-friendly work environment. 

#### 4.2.2. Theoretical Implications

This study also contributes to the theory in a small way. It shows how GM and GCP are intertwined, implying that employees with GM are better at tackling environmental issues. There are rare studies that have examined the relationship between the said variables. In addition, this research has shown that GCPE serves as a mediating link in the relationship between GM and GCP. By first identifying environmental issues and then researching to come up with recommendations for potential solutions, GCPE illuminates the path to achieving GCP. It explains how GM goes about achieving GCP. Employees are more likely to engage in creative activities and achieve GCP if they access rare and non-substitutable resources, such as GM.

### 4.3. Study Limitations and Future Recommendations

There are some limitations to the current study, even though it does make some critical suggestions. The study used a cross-sectional research design. Future studies may adopt a longitudinal research design to validate current research findings further. This study focuses on manufacturing and services firms in emerging economies in Asia (specifically Pakistan); future studies may operationalize this model in multiple countries to generalize the findings. It is also recommended that different contextual factors such as culture, leadership commitment, etc., are included in future studies, since individual and contextual factors can influence the relationship between GM and GCP. A larger share of the respondents, more than 2/3 of the total, were younger individuals. Young respondents may be more “green proficient” than their older counterparts because the greater sensitivity of younger generations to the natural environment may constitute a substantial shortfall of the study. Finally, this study focuses on employees’ green motivation. Future studies are invited to investigate a similar model from the employer perspective.

## 5. Conclusions

Sustainability and environmental concerns have been given a lot of attention in the recent literature by researchers and industrialists. There has been a shift in modern business practices toward environmentally friendly practices, products, and procedures. The current study builds its arguments on the green and CoR theories, which suggest that firms must ensure sustainability at all levels. Employees’ resources, like GM, help them engage in GCP and deal with challenging situations like the achievement of environment-friendly challenges, leading to the accumulation of additional resources. This study’s findings are that employees’ GM significantly predicts the GCP of employees. Moreover, GCPE serves as a partial mediator between both variables.

## Figures and Tables

**Table 1:** Demographic of respondents.

**Particulars**	**Description**	**Value**	**Percentage**
Job Position	Operational staff	123	39.5%
	Junior manager	63	20.2%
	Middle manager	88	28.2%
	Top manager	37	11.9%
Industry	Manufacturing	173	55.63%
	Services	138	44.37%
Gender	Male	203	65.2%
	Female	108	34.7%
Experience	1 month–5 years	121	38.9%
	6 years–10 years	87	27.9%
	11 years–15 years	54	17.3%
	Above 16 years	49	15.7%

**Table 2:** Reliability and validity of the instrument.

**Construct**	**Items**	**Cronbach Alpha**	**Factor Loading Ranges**	**Composite Reliability**	**AVE**
Green Motivation	9	0.826	0.736–0.925	0.876	0.637
Green Creative Processes Engagement	11	0.912	0.754–0.951	0.865	0.656
Green Creative Performance	10	0.891	0.734–0.944	0.943	0.689

Extraction method: Principal Component Analysis Rotation Method: Promax with Kaiser Normalization, loading at 0.40 composite reliability should be ≥0.7 (Molina et al., 2007 [53]). The average variance extracted (AVE) should be ≥0.5 (Molina et al., 2007 [53]).

**Table 3:** Constructs’ discriminant validity.

Construct	GM	GCPE	GCP
GM	***0.798***		
GCPE	0.496	***0.809***	
GCP	0.507	0.459	***0.830***

GM = Green Motivation, GCPE = Green Creative Processes Engagement, GCP = Green Creative Performance. The AVE square root value for each construct is mentioned in bold and italic.

**Table 4:** Model fit measures.

The Goodness of Fit Measures	CMIN/DF	NFI	GFI	AGFI	CFI	TLI	RMSEA	SRMR
Recommended value	≤3 ^1^	≥0.9 ^2^	≥0.9 ^2^	≥0.9 ^2^	≥0.9 ^2^	≥0.9 ^2^	≤0.08 ^3^	≤0.08 ^4^
Measurement model	1.164	0.926	0.911	0.927	0.942	0.954	0.029	0.0352
Structural model	1.191	0.931	0.922	0.951	0.956	0.951	0.034	0.0331

^1^ (Byrne 1989 [57]; Bagozzi and Yi 1988 [58]). ^2^ (Bollen 1986 [59]). ^3^ (Browne and Cudeck 1992 [60]). ^4^ (Hu and Bentler 1998 [61]).

**Table 5:** Results of hypothesis testing.

**Hypothesis**	**Constructs**	**Standardized Estimate**	**Critical Ratio**	***p*** **-Value**	**Decision**
H1	GM → GCP	0.294	2.684	0.003 *	Accepted
H2	GM → GCPE	0.301	2.782	0.003 *	Accepted
H3	GCPE → GCP	0.348	3.673	0.001 **	Accepted
**Mediation**					
H4	GM → GCP	0.226	2.198	0.034 *	Partially Accepted
	GM → GCPE	0.274	2.225	0.171 *	
	GCPE → GCP	0.301	2.994	0.003 *	
**Control Variables**					
Indus. Type → GCP		0.236	2.369	0.028 *	Significant
Industry type → GCPE		0.296	3.102	0.009 *	Significant
Gender → GCP		0.199	2.01	0.048 *	Significant
Gender → GCPE		2.009	2.137	0.021 *	Significant

* *p* 0.05; ** *p* 0.01; GM = Green Motivation, GCPE = Green Creative Processes Engagement, GCP = Green Creative Performance, GC = Green Creativity, GIB = Green Innovative Behavior.

## References

[1] Ahmed M., Guo Q., Qureshi M.A., Raza S.A., Khan K.A., Salam J. (2021). Do Green HR Practices Enhance Green Motivation and Proactive Environmental Management Maturity in Hotel Industry?. Int. J. Hosp. Manag..

[2] Abbas J., Sağsan M. (2019). Impact of Knowledge Management Practices on Green Innovation and Corporate Sustainable Development: A Structural Analysis. J. Clean. Prod..

[3] UNGC (2018). United Nations Global Compact.

[4] Fu Q., Abdul Rahman A.A., Jiang H., Abbas J., Comite U. (2022). Sustainable Supply Chain and Business Performance: The Impact of Strategy, Network Design, Information Systems, and Organizational Structure. Sustainability.

[5] Al-Ghazali B.M., Afsar B. (2021). Retracted: Green Human Resource Management and Employees’ Green Creativity: The Roles of Green Behavioral Intention and Individual Green Values. Corp. Soc. Responsib. Environ. Manag..

[6] Xie Z., Liu X., Najam H., Fu Q., Abbas J., Comite U., Cismas L.M., Miculescu A. (2022). Achieving Financial Sustainability through Revenue Diversification: A Green Pathway for Financial Institutions in Asia. Sustainability.

[7] Abbas J. (2020). Impact of Total Quality Management on Corporate Green Performance through the Mediating Role of Corporate Social Responsibility. J. Clean. Prod..

[8] Luu T.T. (2021). Green Creative Behavior in the Tourism Industry: The Role of Green Entrepreneurial Orientation and a Dual-Mediation Mechanism. J. Sustain. Tour..

[9] Prasetyo P.E., Dzaki F.Z. (2020). Institutional Performance and New Product Development Value Chain for Entrepreneurial Competitive Advantage. Uncertain Supply Chain Manag..

[10] Habib M., Abbas J., Noman R. (2019). Are Human Capital, Intellectual Property Rights, and Research and Development Expenditures Really Important for Total Factor Productivity? An Empirical Analysis. Int. J. Soc. Econ..

[11] Khan S.M., Abbas J. (2022). Mindfulness and Happiness and Their Impact on Employee Creative Performance: Mediating Role of Creative Process Engagement. Think. Ski. Creat..

[12] Mutonyi B.R., Slåtten T., Lien G. (2020). Organizational Climate and Creative Performance in the Public Sector. Eur. Bus. Rev..

[13] Singjai K., Winata L., Kummer T.-F. (2018). Green Initiatives and Their Competitive Advantage for the Hotel Industry in Developing Countries. Int. J. Hosp. Manag..

[14] Abbas J. (2020). Impact of Total Quality Management on Corporate Sustainability through the Mediating Effect of Knowledge Management. J. Clean. Prod..

[15] Kalyar M.N., Ali F., Shafique I. (2021). Green Mindfulness and Green Creativity Nexus in Hospitality Industry: Examining the Effects of Green Process Engagement and CSR. Int. J. Contemp. Hosp. Manag..

[16] Tuan L.T. (2021). Effects of Environmentally-Specific Servant Leadership on Green Performance via Green Climate and Green Crafting. Asia Pac. J. Manag..

[17] Khan H., Abbas J., Kumari K., Najam H. (2022). Corporate Level Politics from Managers and Employees Perspective and Its Impact on Employees’ Job Stress and Job Performance. J. Econ. Adm. Sci..

[18] Junsheng H., Masud M.M., Akhtar R., Rana M.D.S. (2020). The Mediating Role of Employees’ Green Motivation between Exploratory Factors and Green Behaviour in the Malaysian Food Industry. Sustainability.

[19] Kollmuss A., Agyeman J. (2002). Mind the Gap: Why Do People Act Environmentally and What Are the Barriers to pro-Environmental Behavior?. Environ. Educ. Res..

[20] Li W., Bhutto T.A., Xuhui W., Maitlo Q., Zafar A.U., Bhutto N.A. (2020). Unlocking Employees’ Green Creativity: The Effects of Green Transformational Leadership, Green Intrinsic, and Extrinsic Motivation. J. Clean. Prod..

[21] Gilal F.G., Ashraf Z., Gilal N.G., Gilal R.G., Channa N.A. (2019). Promoting Environmental Performance through Green Human Resource Management Practices in Higher Education Institutions: A Moderated Mediation Model. Corp. Soc. Responsib. Environ. Manag..

[22] Kim J.-E. (2019). The Impact of Creative Role Identity and Creative Self-Efficacy on Employee Creativity in the Hotel Business. J. Asian Financ. Econ. Bus..

[23] Jerónimo H.M., Henriques P.L., de Lacerda T.C., da Silva F.P., Vieira P.R. (2020). Going Green and Sustainable: The Influence of Green HR Practices on the Organizational Rationale for Sustainability. J. Bus. Res..

[24] Ahsan M.U., Nasir M., Abbas J. (2020). Examining the Causes of Plastic Bags Usages and Public Perception about Its Effects on the Natural Environment. Int. J. Acad. Res. Bus. Soc. Sci..

[25] Ojo A.O., Raman M., Downe A.G. (2019). Toward Green Computing Practices: A Malaysian Study of Green Belief and Attitude among Information Technology Professionals. J. Clean. Prod..

[26] Ali F., Ashfaq M., Begum S., Ali A. (2020). How “Green” Thinking and Altruism Translate into Purchasing Intentions for Electronics Products: The Intrinsic-Extrinsic Motivation Mechanism. Sustain. Prod. Consum..

[27] Hughes D.J., Lee A., Tian A.W., Newman A., Legood A. (2018). Leadership, Creativity, and Innovation: A Critical Review and Practical Recommendations. Leadersh. Q..

[28] Mahmood M., Uddin M.A., Fan L. (2019). The Influence of Transformational Leadership on Employees’ Creative Process Engagement: A Multi-Level Analysis. Manag. Decis..

[29] George G., Schillebeeckx S.J., Liak T.L. (2018). The Management of Natural Resources: An Overview and Research Agenda. Managing Natural Resources.

[30] Uddin M.M.M. (2020). What Are the Dynamic Links between Agriculture and Manufacturing Growth and Environmental Degradation? Evidence from Different Panel Income Countries. Environ. Sustain. Indic..

[31] Eckersley R. (2010). Green Theory.

[32] Edgeman R., Eskildsen J. (2014). Modeling and Assessing Sustainable Enterprise Excellence. Bus. Strategy Environ..

[33] Hobfoll S.E. (2011). Conservation of Resources Theory: Its Implication for Stress, Health, and Resilience. The Oxford Handbook of Stress, Health, and Coping.

[34] (2016). UNESCO. Towards 2030: Creativity Matters for Sustainable Development—Google Search.

[35] UNPRME. https://www.unprme.org/what-we-do.

[36] Kumari K., Ali S.B., un Nisa Khan N., Abbas J. (2021). Examining the Role of Motivation and Reward in Employees’ Job Performance Through Mediating Effect of Job Satisfaction: An Empirical Evidence. Int. J. Organ. Leadersh..

[37] Song M., Yang M.X., Zeng K.J., Feng W. (2020). Green Knowledge Sharing, Stakeholder Pressure, Absorptive Capacity, and Green Innovation: Evidence from Chinese Manufacturing Firms. Bus. Strategy Environ..

[38] Bos-Nehles A., Renkema M., Janssen M. (2017). HRM and Innovative Work Behaviour: A Systematic Literature Review. Pers. Rev..

[39] Yusoff Y.M., Nejati M., Kee D.M.H., Amran A. (2020). Linking Green Human Resource Management Practices to Environmental Performance in Hotel Industry. Glob. Bus. Rev..

[40] Cheng C., Yang M. (2019). Creative Process Engagement and New Product Performance: The Role of New Product Development Speed and Leadership Encouragement of Creativity. J. Bus. Res..

[41] Cheung S.Y., Huang E.G., Chang S., Wei L. (2020). Does Being Mindful Make People More Creative at Work? The Role of Creative Process Engagement and Perceived Leader Humility. Organ. Behav. Hum. Decis. Process..

[42] Zhang X., Bartol K.M. (2010). The Influence of Creative Process Engagement on Employee Creative Performance and Overall Job Performance: A Curvilinear Assessment. J. Appl. Psychol..

[43] Bin Saeed B., Afsar B., Shahjehan A., Imad Shah S. (2019). Does Transformational Leadership Foster Innovative Work Behavior? The Roles of Psychological Empowerment, Intrinsic Motivation, and Creative Process Engagement. Econ. Res.-Ekon. Istraživanja.

[44] Reiter-Palmon R. (2021). Leading for Team Creativity: Managing People and Processes. Creative Success in Teams.

[45] Zhang W., Xu F., Wang X. (2020). How Green Transformational Leadership Affects Green Creativity: Creative Process Engagement as Intermediary Bond and Green Innovation Strategy as Boundary Spanner. Sustainability.

[46] Guay F., Vallerand R.J., Blanchard C. (2000). On the Assessment of Situational Intrinsic and Extrinsic Motivation: The Situational Motivation Scale (SIMS). Motiv. Emot..

[47] Soda G., Stea D., Pedersen T. (2019). Network Structure, Collaborative Context, and Individual Creativity. J. Manag..

[48] Scott S.G., Bruce R.A. (1994). Determinants of Innovative Behavior: A Path Model of Individual Innovation in the Workplace. Acad. Manag. J..

[49] Hair J.F., Anderson R.E., Tatham R.L., Black W.C. (2010). Multivariate Data Analysis.

[50] Kaiser H.F., Rice J. (1974). Little Jiffy, Mark Iv. Educ. Psychol. Meas..

[51] Abbas J. (2020). HEISQUAL: A Modern Approach to Measure Service Quality in Higher Education Institutions. Stud. Educ. Eval..

[52] Hinkin T.R. (1998). A Brief Tutorial on the Development of Measures for Use in Survey Questionnaires. Organ. Res. Methods.

[53] Molina L.M., Lloréns-Montes J., Ruiz-Moreno A. (2007). Relationship between Quality Management Practices and Knowledge Transfer. J. Oper. Manag..

[54] Awang Z. (2012). A Handbook on Structural Equation Modeling Using AMOS.

[55] Fornell C., Larcker D.F. (1981). Evaluating Structural Equation Models with Unobservable Variables and Measurement Error. J. Mark. Res..

[56] Kaynak H. (2003). The Relationship between Total Quality Management Practices and Their Effects on Firm Performance. J. Oper. Manag..

[57] Byrne B.M. (1989). A Primer of LISREL: Basic Applications and Programming for Confirmatory Factor Analytic Models.

[58] Bagozzi R.P., Yi Y. (1988). On the Evaluation of Structural Equation Models. J. Acad. Mark. Sci..

[59] Bollen K.A. (1986). Sample Size and Bentler and Bonett’s Nonnormed Fit Index. Psychometrika.

[60] Browne M.W., Cudeck R. (1992). Alternative Ways of Assessing Model Fit. Sociol. Methods Res..

[61] Hu L., Bentler P.M. (1998). Fit Indices in Covariance Structure Modeling: Sensitivity to Underparameterized Model Misspecification. Psychol. Methods.

[62] Abbas J., Dogan E. (2022). The Impacts of Organizational Green Culture and Corporate Social Responsibility on Employees’ Responsible Behaviour towards the Society. Environ. Sci. Pollut. Res..

[63] Li C.-R., Yang Y., Lin C.-J., Xu Y. (2021). Within-Person Relationship between Creative Self-Efficacy and Individual Creativity: The Mediator of Creative Process Engagement and the Moderator of Regulatory Focus. J. Creat. Behav..

